# Correlation between serum bilirubin, blood uric acid, and C-reactive protein and the severity of chronic obstructive pulmonary disease

**DOI:** 10.1186/s41043-024-00593-5

**Published:** 2024-07-08

**Authors:** Tingting Zhao, Tian Lv

**Affiliations:** 1 Department of Respiratory and Critical Care Medicine, Zhuji People’s Hospital of Zhejiang Province, Zhuji Affiliated Hospital of Wenzhou Medical University, No. 9 Jianmin Road, Taozhu Street, Zhuji, Zhejiang 311800 China; 2 Department of Neurology, Zhuji People’s Hospital of Zhejiang Province, Zhuji Affiliated Hospital of Wenzhou Medical University, Zhuji, 311800 China

**Keywords:** Chronic obstructive pulmonary disease, Serum bilirubin, Blood uric acid, C-reactive protein

## Abstract

To explore the correlation between serum bilirubin, blood uric acid, and C-reactive protein (CRP) and the severity of chronic obstructive pulmonary disease (COPD). Methods: Patients with COPD who were admitted to our hospital between March 2020 and March 2023 were retrospectively studied. Based on whether their condition progressed to the acute exacerbation stage, they were divided into an exacerbation group (100 cases) and a stability group (100 cases). The clinical data from both groups were analysed to assess the correlations between serum bilirubin, blood uric acid, CRP, and the severity of COPD. Results: Univariate analysis indicated significant differences in the neutrophil-to-lymphocyte ratio (*t* = 5.678, *P* < 0.05), α-hydroxybutyrate dehydrogenase (*t* = 5.862, *P* < 0.05), total bilirubin (*t* = 4.341, *P* < 0.05), direct bilirubin (*t* = 5.342, *P* < 0.05), indirect bilirubin (*t* = 5.452, *P* < 0.05), blood uric acid (*t* = 4.698, *P* < 0.05), and CRP (*t* = 4.892, *P* < 0.05) between the two groups. Multivariate analysis revealed that total bilirubin, blood uric acid, and CRP were positively correlated with exacerbations of COPD (regression coefficients were 0.413, 0.354, and 0.356, respectively; *P* < 0.05). The evaluation of predictive value showed that the combined predictive value of these three indicators was the highest, with an AUC of 0.823 (95% CI: 0.754–0.911). Conclusion: Serum bilirubin, blood uric acid, and CRP levels are elevated in patients with acute exacerbations of COPD (AECOPD), showing good consistency in predicting the occurrence of AECOPD. The combined diagnostic value of these three indicators is greater than that of any single indicator, providing a reference for the early clinical prediction of AECOPD.

## Introduction

Chronic obstructive pulmonary disease (COPD) is a heterogeneous lung condition characterised by chronic respiratory symptoms (dyspnoea, cough, sputum production) due to abnormalities of the airways (bronchitis, bronchiolitis) and/or alveoli (emphysema) that cause persistent, often progressive, airflow obstruction [1]. In the course of COPD, recurrent acute attacks often result in poor prognosis. COPD is highly heterogeneous, presenting various phenotypes. Clinically, it can be divided into stable and acute exacerbation stages, with transitions between them. Patients in the stable stage can progress to acute exacerbation of COPD (AECOPD) under the influence of several factors, such as infections, characterised by symptoms such as dyspnoea, cough, and expectoration exceeding daily fluctuations [2], indicating that the original respiratory symptoms have worsened. This requires further adjustment of the treatment regimen to control the disease [3]. As of 2017, there were 384 million people worldwide with COPD, with the numbers rising due to ageing, air pollution, and increasing smoking rates; the incidence of COPD has exceeded 11% [4]. National data indicate that COPD is the third leading cause of disease-related death, after ischemic heart disease and stroke [5]. Its high morbidity and mortality rates pose a substantial burden on individuals and society. Acute exacerbations are the primary reason for these outcomes. Therefore, clinicians must identify changes in patients with COPD early and provide prompt treatment by actively searching for potential biomarkers to predict the onset of acute exacerbations and assess disease.

The pathogenesis of COPD is complex, involving intertwined mechanisms of airway inflammation and an imbalance between oxidation and antioxidation, which play a major role. Recent studies have confirmed the substantial impact of oxidative stress on the onset, progression, and prognosis of COPD, contributing to increased morbidity and mortality [6]. Blood uric acid, the end product of purine metabolism, has long been regarded as a useless metabolite. However, recent findings suggest that blood uric acid possesses both oxidising and antioxidising properties, and excessive levels can aggravate systemic inflammation and damage endothelial cells [7, 8]. High uric acid levels can promote oxidative stress in adipocytes, endothelial cells, and vascular smooth muscle cells, contributing to oxidative stress in COPD and exacerbating the disease. Serum bilirubin, a degradation product of heme, is a strong endogenous antioxidant with anti-inflammatory properties. It combats oxidative damage by binding to albumin, which then neutralises lipid peroxidation free radicals, helping control oxidative stress [9]. Studies indicate that an increase in serum bilirubin within the physiological range can exert a strong antioxidant effect and is inversely correlated with the incidence of COPD and lung cancer, suggesting a protective role in some respiratory diseases [10]. C-reactive protein (CRP), a marker of acute inflammation, is a sensitive indicator of the body’s inflammatory status. A study [11] shown that CRP levels in patients with COPD are not only higher than in healthy individuals but also positively correlate with the severity of COPD. Further large-scale studies have confirmed that CRP levels are elevated in patients with COPD compared with healthy individuals and spike during AECOPD [12], exceeding levels found in stable periods and in healthy individuals. Its level can predict the occurrence and outcomes of AECOPD [13, 14]. Although research has explored the association between each of these indicators and the severity of COPD, studies examining the combined impact of serum bilirubin, blood uric acid, and CRP on COPD severity are limited. Therefore, this study utilises these three biomarkers to investigate their relationship with the severity of COPD.

## Research participants and methods

### Research participants

Patients with COPD admitted to the respiratory medicine department of XX Hospital between March 2020 and March 2023 were retrospectively enrolled and divided into an exacerbation group (100 cases) and a stability group (100 cases) based on the progression of their COPD to the acute exacerbation stage. The inclusion criteria included the following: (1) diagnosis of COPD meeting international standards (FEV1/FVC < 0.7) [15]; (2) diagnostic criteria for AECOPD aligned with the 2017 Consensus of Chinese Experts on the Diagnosis and Treatment of Acute Exacerbation of Chronic Obstructive Pulmonary Disease [16]. The exclusion criteria included the following: (1) biliary tract-related diseases (acute pancreatitis, liver cirrhosis, severe hepatitis, abdominal tumour with infection); (2) blood-related diseases (polycythaemia, leukaemia, multiple myeloma, pernicious anaemia); (3) primary or secondary gout, renal insufficiency; (4) coronary atherosclerotic heart disease, hypertension, and acute cardiovascular and cerebrovascular diseases; (5) patients with bronchiectasis, interstitial lung disease, lung malignancy, and tuberculosis; (6) infectious system diseases (trauma, digestive system infection, urinary system infection, skin and soft tissue infection).

### Methods

Venous blood was collected from all patients within the first 24 h of admission. Serum bilirubin and blood uric acid levels were analysed using a biochemical analyser (AU5800 from Maccura Biotechnology Co. Ltd.). The normal reference range for serum bilirubin was 5.1–28 µmol/L, and for blood uric acid was 210–430 µmol/L (men) and 150–360 µmol/L (women). CRP levels were measured by an automatic specific protein analyser (PA200 from Shenzhen Genrui Company), with a normal value of ≤ 10 mg/L. Complete blood cell counts and 5-category examinations were performed using an automatic blood cell analyser (Abbott-RUBY), and lung function was assessed using the MEDGRAPHICS lung function test system (ELITEDL from McAfee Company).

### Data collection

General and serological data were collected from both groups. General data included age, gender, height, body mass index (BMI), history of stroke, diabetes, smoking, hypertension, drinking, cerebrovascular disease, cancer, hyperlipidaemia, and education. Serological data encompassed alanine aminotransferase (ALT), aspartate aminotransferase (AST), creatinine, serum sodium, haemoglobin (HB), neutrophil-to-lymphocyte ratio (NLR), α-hydroxybutyrate dehydrogenase (α-HBDH), total bilirubin, direct bilirubin, indirect bilirubin, blood uric acid, and CRP.

### Statistical analysis

Statistical analysis was performed using SPSS 26.0. Data normally distributed were expressed as x ± s. Paired data were analysed using the paired t-test, and variance analysis was employed to compare multiple groups. Count data were presented as frequency or rate and analysed with the χ^2^ test. The rank sum test was used to compare graded variables between groups. Multivariate linear regression was applied for multivariate analysis, with a *P*-value of < 0.05 considered statistically significant.

## Results

### Comparison of clinical data between the two groups

**Fig. 1:**
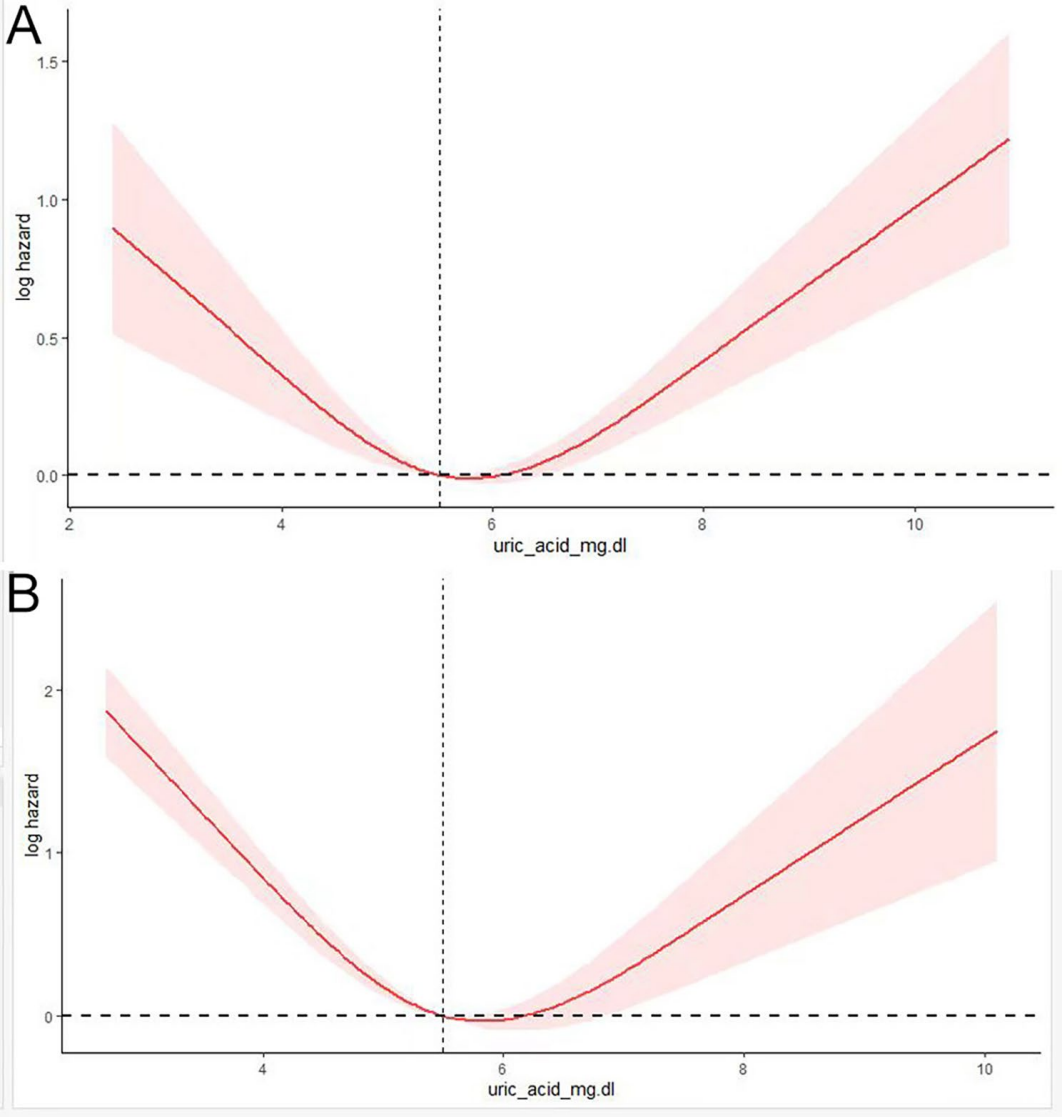
Multivariable- adjusted HRs for (**A**) all- cause and (**B**) Chronic lower respiratory diseases by uric acid level. Sex, Dm, Hypertension, Hyperlipidemia, cerebrovascular disease, cancer, stroke, age, lymphocyte, creatinine, Alt, NLR, BMI.

In the exacerbation group, there were 57 men and 43 women, with smokers comprising 53% of the group. The average age was 71.34 ± 11.51 years, and the BMI was 21.32 ± 6.67. In the control group, there were 55 men and 45 women, with 59% smokers, and the average age was 73.41 ± 11.35 years. There were no significant differences in age, gender, height, BMI, history of stroke, diabetes, smoking, hypertension, drinking, cerebrovascular disease, cancer, hyperlipidaemia, education level, ALT, AST, creatinine, serum sodium, and HB between the two groups (*P* > 0.05). Significant differences were observed in the NLR, α-HBDH, total bilirubin, direct bilirubin, indirect bilirubin, blood uric acid, and CRP (*P* < 0.05), as illustrated in Table 1; Fig. 1.

**Table 1:** Comparison of general data between the two groups

Item		Exacerbation group (*n* = 100)	Stability group (*n* = 100)	χ^2^/t/Z value	*P* value
Gender (male/female)		57/43	55/45	0.081	> 0.05
Age (years old, x ± s))		71.34 ± 11.51	73.41 ± 11.35	0.931	> 0.05
Height (cm, x ± s)		163.44 ± 11.45	156.58 ± 12.97	0.992	> 0.05
Body mass index (kg/m^2^, x ± s)		21.32 ± 6.67	21.79 ± 7.13	1.113	> 0.05
History of stroke (case)		11	9	0.222	> 0.05
History of diabetes mellitus (case)		19	25	1.049	> 0.05
Smoking history (case)		53	59	0.731	> 0.05
History of hypertension (case)		31	26	0.613	> 0.05
Drinking history (case)		35	41	0.764	> 0.05
History of cerebrovascular disease (case)		22	24	0.113	> 0.05
Cancer history (case)		21	19	0.125	> 0.05
Hyperlipidemia (case)		22	31	2.079	> 0.05
Alanine aminotransferase (U/L, x ± s)		21.45 ± 6.23	18.67 ± 5.56	0.628	> 0.05
Aspartate aminotransferase (U/L, x ± s)		15.23 ± 4.62	16.32 ± 5.57	0.728	> 0.05
Creatinine (µmol/L, x ± s)		61.68 ± 8.68	73.56 ± 10.79	0.768	> 0.05
Serum sodium (mmol/L, x ± s)		139.56 ± 5.51	141.68 ± 5.23	0.567	> 0.05
Hemoglobin (g/L, x ± s)		134.78 ± 10.35	141.67 ± 12.89	0.314	> 0.05
Neutrophil to lymphocyte ratio		14.36 ± 9.21	6.22 ± 4.41	5.678	< 0.05
α-hydroxybutyrate dehydrogenase (U/L, x ± s)		232.73 ± 52.52	153.2 ± 51.43	5.862	< 0.05
Total bilirubin (µmol/L, x ± s)		11.87 ± 5.32	8.12 ± 3.97	4.341	< 0.05
Direct bilirubin (µmol/L, x ± s)		4.53 ± 2.53	2.75 ± 1.34	5.342	< 0.05
Indirect bilirubin (µmol/L, x ± s)		7.13 ± 3.53	5.23 ± 2.34	5.452	< 0.05
Blood uric acid (µmol/L, x ± s)		269.31 ± 73.41	202.65 ± 78.54	4.698	< 0.05
C-reactive protein (mg/L, x ± s)		17.62 ± 5.26	7.14 ± 2.04	4.892	< 0.05
Education level (case)	Primary school	29	32	-1.044	> 0.05
	Middle school / technical secondary school	45	50		
	Junior college or above	26	18		

### Multivariate analysis of frequent exacerbation of chronic obstructive pulmonary disease

Multivariate linear regression analysis was employed to explore the relationships between the NLR, α-HBDH, total bilirubin, blood uric acid, and CRP in relation to the exacerbation of COPD. The findings indicated that total bilirubin, blood uric acid, and CRP were significantly associated with COPD exacerbation (regression coefficients were 0.413, 0.354, and 0.356, respectively; *P* < 0.05). Higher levels of these markers were linked with an increased likelihood of COPD exacerbation, as presented in Table 2.

**Table 2:** Multivariate regression analysis of frequent exacerbation of chronic obstructive pulmonary disease

Item	Regression coefficient	Standard error	Wald	*P* value
Constant term	2.712	0.531	8.381	0.001
Neutrophil to lymphocyte ratio	0.134	0.045	1.234	0.824
α-hydroxybutyrate dehydrogenase	0.224	0.056	1.008	0.925
Total bilirubin	0.413	0.078	5.003	0.001
Blood uric acid	0.354	0.024	4.423	0.001
C-reactive protein	0.356	0.015	4.535	0.001

### Predictive value of total bilirubin, blood uric acid, C-reactive protein, and combined diagnosis for exacerbation of chronic obstructive pulmonary disease

Total bilirubin, blood uric acid, and CRP each showed predictive value for the exacerbation of COPD (*P* < 0.05). The area under the curve (AUC) for total bilirubin in predicting COPD exacerbation was 0.607 (95% CI: 0.531–0.736); for blood uric acid, the AUC was 0.734 (95% CI: 0.642–0.821); and for CRP, the AUC was 0.613 (95% CI: 0.531–0.745). However, the combined predictive value of these three biomarkers was the highest, with an AUC of 0.823 (95% CI: 0.754–0.911). The AUC for the combined prediction of COPD exacerbation was significantly higher than that of the individual biomarkers, with Z-values of 2.431, 2.453, and 3.412, respectively (*P* < 0.05). Details are provided in Table 3.

**Table 3:** Predictive value of total bilirubin, serum uric acid, C-reactive protein and combined diagnosis for exacerbation of chronic obstructive pulmonary disease

Item	Accuracy	Sensitivity	Specificity	AUC	95%CI
Total bilirubin	0.854	0.842	0.801	0.607	0.531 ~ 0.736
Blood uric acid	0.813	0.831	0.798	0.734	0.642 ~ 0.821
C-reactive protein	0.841	0.812	0.831	0.613	0.531 ~ 0.745
Combined prediction	0.921	0.911	0.905	0.823	0.754 ~ 0.911

## Discussion

In terms of patient impact, AECOPD leads to a further decline in quality of life, increases the economic burden on patients, and can even result in death in severe cases. Therefore, in the overall treatment process of COPD, the prevention and management of AECOPD are crucial, and identifying patients with AECOPD as early as possible is vital. To date, scholars worldwide have classified AECOPD according to GOLD guidelines based on the exacerbation of cough, sputum, and asthma symptoms and the presence of these three symptoms. They have then decided whether to use antibiotics for treatment. Although some studies [17–19] have provided more detailed evaluation criteria for changes in respiratory symptoms in patients with AECOPD, these criteria are largely similar and are all based on the subjective symptoms of patients. Since patients with COPD exhibit symptoms of cough, phlegm, and asthma throughout the course of the disease, each patient has a different sensitivity to symptoms, and the severity of symptoms can also be affected by psychological factors. Therefore, judging AECOPD based solely on patients’ subjective symptoms is not accurate. To date, the pathogenesis of COPD has not been fully elucidated, and the recognised mechanisms include airway inflammation, oxidative stress, an imbalance between protease and antiprotease, and enhanced cholinergic nerve activity in the airways; the pathogenic factors are diverse, including bacterial and viral infections, air pollution, cold currents, cigarette smoke, and solid fuels. In terms of AECOPD occurrence, infection, deviation from baseline treatment, and resumption of smoking are considered the main factors that exacerbate COPD and oxidative stress. Therefore, given that the pathogenesis is not completely understood finding relevant biomarkers to predict exacerbation of COPD is of great importance.

Serum bilirubin is the downstream product of human heme metabolism and accounts for one-third of the total antioxidant capacity of the body. It not only serves an antioxidant role but also helps resist inflammation and prevent apoptosis. In the process of bilirubin metabolism and antioxidation, the key enzyme is heme oxygenase (HO), which is crucial for balancing the synthesis and catabolism of bilirubin [20]. HO-1, an isozyme of HO and a stress-reactive protein, is an inducible form that sees substantially increased serum levels during oxidative stress, thereby inducing higher bilirubin levels. Because patients with COPD experience persistent airflow restriction, leading to chronic hypoxia, the balance between oxidants and antioxidants is disrupted, consuming large amounts of antioxidants and producing large amounts of oxides. HO-1 plays a role in the onset and progression of COPD. In cases where patients have severe COPD, the expression of HO-1 in lung macrophages and bronchoalveolar lavage fluid is diminished. Studies using a mouse model show that overexpression of HO-1, mediated by adenovirus, can inhibit the development of emphysema induced by trypsin, suggesting that overexpression of HO-1 can impede the progression of emphysema [21]. This study’s results indicate that patients with COPD with added recombinant bilirubin have higher levels than those in the stability group, a finding inconsistent with other domestic and international studies. The increase in bilirubin during AECOPD is speculated to be compensatory, whereas in the stable phase of COPD, bilirubin is consumed as an antioxidant.

Blood uric acid can interact with other antioxidants such as superoxide dismutase, ascorbic acid, and tetrahydrobiopterin, acting as a free radical scavenger and iron chelating agent to reduce oxidative stress. A long-term oxidative stress environment can decrease blood uric acid levels, which are positively correlated with antioxidant capacity [22]. During COPD, when the balance between oxidation and antioxidation is disturbed, the antioxidant role of blood uric acid in patients with COPD is substantially diminished compared with healthy individuals [23]. The results of this study indicate that blood uric acid levels are higher in the COPD exacerbation group compared with the stability group, a finding that contradicts both domestic and international studies. This study speculates that similar to bilirubin, during AECOPD, the body may protect itself from oxidative damage by elevating blood uric acid levels under stress to enhance its antioxidant role. However, the persistent imbalance between oxidation and antioxidation in patients with COPD diminishes the compensatory ability of antioxidants and depletes uric acid to a low level.

C-reactive protein, as a marker of systemic inflammatory response, is an acute-phase protein that increases when inflammation occurs in the body. Many studies have confirmed that CRP levels are higher in the stable stage of COPD than in normal individuals because, although specific inflammation is less severe than in the acute exacerbation stage, non-specific inflammation still persists. This non-specific inflammation puts the body under stress, inducing the production of CRP, which activates the complement system and phagocytes to clear apoptotic and necrotic cells, thereby protecting the host through a protective compensatory mechanism [24–26]. The results of this study indicate that levels of CRP in patients with COPD with added recombinant CRP are higher than those in the stability group, consistent with findings both domestically and internationally. This supports the hypothesis that CRP may be linked to the onset of COPD and suggests that monitoring changes in CRP can help predict the occurrence of AECOPD.

Of course, this study has some limitations. First, being a single-centre study, ensuring consistent baseline characteristics when grouping and comparing cohorts is challenging, and patients may have other complications that could affect their prognosis. Second, serum bilirubin levels can be influenced by many factors, such as smoking [27], gender, age, and race; similarly, baseline levels of blood uric acid are easily affected by the frequency of smoking [28], gender, age, dietary habits, purine intake, race, and nutritional status. C-reactive protein levels are also affected by factors such as smoking, pathogen types, medications, and individual qualities. Finally, due to time and manpower constraints, the sample size is small, and the representativeness of the samples may be poor, necessitating further exploration in future studies aimed at larger samples and multi-centre collaborations.

## Conclusion

In conclusion, serum bilirubin, blood uric acid, and CRP levels are generally observed to be elevated in patients with exacerbations of COPD, and they appear to show consistent potential in predicting the occurrence of AECOPD. The combined diagnostic value of these three indicators tends to be greater than that of any single indicator, suggesting that they could provide a valuable reference for the early clinical prediction of AECOPD.

## Data Availability

All data generated or analyzed during this study are included in this article.
